# Antibacterial activity of thymoquinone derivative

**DOI:** 10.1186/s13104-023-06523-8

**Published:** 2023-10-06

**Authors:** Mohammad Yasin Mohammad, Haroon M. Haniffa, M. Iqbal Choudhary

**Affiliations:** 1 Faculty of Pharmacy, Aqaba University of Technology, Aqaba, 11191 Jordan; 2 Department of Chemical Sciences, Faculty of Applied Sciences, South Eastern University, Oluvil, Sri Lanka; 3 H. E. J, Research Institute of Chemistry, International Center for Chemical and Biological Sciences, University of Karachi, Karachi, Pakistan

**Keywords:** Monoterpenoid, Pathogenic bacteria, *Bacillus subtilis*, *Escherichia coli*

## Abstract

Natural products such as terpenoidal compounds have been extremely tested against pathogenic bacteria. Researches are frequently carried out to find out new natural, semisynthetic and synthetic antibacterial agents due to problems of resistance. Thymoquinone derivative was obtained in our previous study and the current research is a continuation. The antibacterial activity of a monoterpenoid; thymoquinone derivative, 5-isopropyl-2-methyloxepine-1-one (**1**) has been evaluated for the first time by following the Agar cup bioassay method employed. The bacterial strains used in this study were *Escherichia coli* and *Bacillus subtilis*. Compound **1** showed moderate activity against Gram-positive organism; *B*. *subtilis* and good activity against Gram-negative species; *E*. *coli* with zones of inhibition (ZOI) 10.0 ± 0.2 mm and 11.0 ± 0.2 mm against *E*. *coli* and *B*. *subtilis*, respectively, and in comparison with antibiotic, imipenem. The zones of inhibition were calculated as the mean of the triplicate. The antibacterial activity of thymoquinone derivative **1** could be explained by the presence of unsaturated lactone.

## Introduction

Thymoquinone (2-Isopropyl-5-methyl-[1,4]benzoquinone, C_10_H_12_O_2_), a monoterpenoid isolated from the seeds of *Nigella sativa*, has been shown to have antioxidant [1], analgesic [2], anticonvulsant effects [3] and anti-bacterial activity [4].

The derivative of thymoquinone, 5-isopropyl-2-methyloxepine-1-one (**1**), was obtained for the first time from our previous work on biotransformation of thymoquinone using *Aspergillus niger* [5], and the current study is a continuation to our research.

In continuation of the biotransformation study on thymoquionone [5], we reported the antibacterial activity of thymoquinone derivative **1** on bacterial strains; *Escherichia coli* and *Bacillus subtilis*.

Antibacterial resistance which occurs naturally, is one of the biggest problems to global health. It can affect any patient, of any age, in any country [6]. In addition, there are problems with the patient staying in the hospital for a longer period of time, high treatment costs, and increased mortality rate [7]. Antibacterial drug resistance by *E. coli* and *B. subtilis* is one of the challenges facing the medical sector [8, 9], so in this study, we evaluated the antibacterial activity against both *E. coli* and *B. subtilis* in order to find out a new lead as a starting point for developing it as antibacterial drug.

## Experimental

### Antibacterial test experiment

The standard strains of bacteria used for this study were *Bacillus subtilis* ATCC 6633 and *Escherichia coli* ATCC 12,022. The bacteria were grown on Muller Hinton II agar (Oxoid) and incubated at 35 °C while slants were prepared and stored at − 4 °C for further studies. Single colony of the test organism was incubated in to nutrient broth for 18 h and diluted 10^5^ folds to obtain approximately 10^6^ colony forming units (cfu) per mL of culture suspension. 100 µL of bacterial suspension was transferred into sterile soft agar tube and properly missed. This was aseptically transmitted into sterile solidified nutrient agar plate to form lawn and gently swirl to ensure even distribution of the test culture and allowed to properly solidify. Sterile cork borer was used to make wells on solidified medium and properly labeled. 1 mg/1 mL of test compound was dissolved in DMSO with 100 µL of test compound in respective well plate according to bacterial culture in triplicates. Reference antibiotic drug and DMSO were added to separate wells as positive and negative control. The plates were allowed to rest for 30 min for proper diffusion prior to incubation at 37 °C for 24 h. The zones of inhibition were measured in mm with transparent ruler and the average was calculated as the mean of the triplicate.

## Results and discussion

The antibacterial activity of thymoquinone derivative, 5-isopropyl-2-methyloxepine-1-one (**1**) has been evaluated for the first time (Table 1) by following the Agar cup bioassay method employed [10]. Compound **1** has shown zones of inhibition (ZOI) 10.0 ± 0.2 mm and 11.0 ± 0.2 mm against *Escherichia coli* and *Bacillus subtilis*, respectively, and in comparison with antibiotic, imipenem. The zones of inhibition were calculated as the mean of the triplicate.

**Table 1:** Inhibition zone diameter (mm) of compound 1*

Compound (100 µg/mL)	Structure	Gram positive *Bacillus subtilis*	Gram negative *Escherichia coli*
**1**		11.0 ± 0.2	10.0 ± 0.2
DMSO	–	–	–
Imipenem	–	29.0 ± 0.1	24.0 ± 0.2

Compound with a concentration of 100 µg/mL was tested against the organisms

Results were recorded after 24 h of treatment and inhibitory zone diameters were measured in mm

–: no inhibition

*Values are mean of triplicate

The antibacterial activity of compound **1** could be explained by the presence of unsaturated lactone [11], since natural products of lactone functionality including terpenoidal lactones showed good anti-bacterial activities against various Gram-positive and Gram-negative organisms [12]. In support of this, for instance, the sesquiterpenoidal bicyclic lactone; costunolide exhibited potent activities against *Mycobacterium tuberculosis* and *Pseudomonas aeruginosa* with MIC 12.5 mg/L and 0.5 mg/mL, respectively [13, 14]. Similarly, the sesquiterpenoidal lactone; partenolide showed potent inhibitions of bacterial growth against *Staphylococcus aureus* and *P. aeruginosa* with MIC 0.08 mg/mL and 0.531 mg/mL, respectively [14].

The antibacterial activities of compound **1** against both *E. coli* and *B. subtilis* were good and not weak compared to the antibiotic; imipenem, and this explains the importance of its lactone functionality to give good activities. However, compound **1** is a lead compound and might be further developed to become an antibacterial agent.

### Limitations

- Insufficient sample amount for tests on other bacterial strains.
- Limited available bacterial strains in our research laboratories.
- Lack of available techniques to evaluate the MIC in this study.

## Conclusion

Compound **1** is a lead compound that showed significant zone of inhibition against both Gram-positive organism; *Bacillus subtilis* and Gram-negative species; *Escherichia coli*, and in comparison with antibiotic; imipenem. In conclusion, metabolite **1** can be further developed to be an antibacterial drug.

## Data Availability

Not applicable.
